# Epigenetic Marks as Predictors of Metabolic Response to Bariatric Surgery: Validation from an Epigenome Wide Association Study

**DOI:** 10.3390/ijms241914778

**Published:** 2023-09-30

**Authors:** Carolina Gutiérrez-Repiso, Antonio Cantarero-Cuenca, Andrés González-Jiménez, Teresa Linares-Pineda, Nerea Peña-Montero, Luis Ocaña-Wilhelmi, Francisco J. Tinahones, Sonsoles Morcillo

**Affiliations:** 1 Unidad de Gestión Clínica de Endocrinología y Nutrición, Hospital Clínico Universitario Virgen de la Victoria, Campus de Teatinos s/n, 29010 Málaga, Spain; teresamaria712@gmail.com (T.L.-P.); nereamontero4@gmail.com (N.P.-M.); fjtinahones@uma.es (F.J.T.); 2 Instituto de Investigación Biomédica de Málaga y Plataforma en Nanomedicina-IBIMA Plataforma BIONAND, Campus de Teatinos s/n, 29010 Málaga, Spain; 3 Centro de Investigación Biomédica en Red de Fisiopatología de la Obesidad y la Nutrición (CIBERobn), Instituto de Salud Carlos III, 28029 Madrid, Spain; 4 ECAI Bioinformática, Instituto de Investigación Biomédica de Málaga y Plataforma en Nanomedicina-IBIMA Plataforma BIONAND, 29590 Málaga, Spain; acantarero@ibima.eu (A.C.-C.); bioinformatica@ibima.eu (A.G.-J.); 5 Unidad de Gestión Clínica de Cirugía General, Digestiva y Trasplantes, Hospital Universitario Virgen de la Victoria, 29590 Málaga, Spain; luisowilhelmi@uma.es; 6 Departamento de Especialidades Quirúrgicas, Bioquímica e Inmunología, Universidad de Málaga, 29590 Málaga, Spain; 7 Departamento de Medicina y Dermatología, Universidad de Málaga, 29590 Málaga, Spain

**Keywords:** bariatric surgery, methylation, metabolic syndrome

## Abstract

Little is known about the potential role of epigenetic marks as predictors of the resolution of obesity-related comorbidities after bariatric surgery. In this study, 20 patients were classified according to the metabolic improvement observed 6 months after sleeve gastrectomy, based on the diagnosis of metabolic syndrome, into responders if metabolic syndrome reversed after bariatric surgery (*n* = 10) and non-responders if they had metabolic syndrome bariatric surgery (*n* = 10). Blood DNA methylation was analyzed at both study points using the Infinium Methylation EPIC Bead Chip array-based platform. Twenty-six CpG sites and their annotated genes, which were previously described to be associated with metabolic status, were evaluated. Cg11445109 and cg19469447 (annotated to Cytochrome P450 2E1 (*CYP2E1*) gene) were significantly more hypomethylated in the responder group than in the non-responder group at both study points, whilst cg25828445 (annotated to Nucleolar Protein Interacting With The FHA Domain Of MKI67 Pseudogene 3 (*NIFKP3*) gene) showed to be significantly more hypermethylated in the non-responder group compared to the responder group at both study points. The analysis of the methylation sites annotated to the associated genes showed that *CYP2E1* had 40% of the differentially methylated CpG sites, followed by Major Histocompatibility Complex, Class II, DR Beta 1 (*HLA-DRB1*) (33.33%) and Zinc Finger Protein, FOG Family Member 2 (*ZFPM2*) (26.83%). Cg11445109, cg19469447 and cg25828445 could have a role in the prediction of metabolic status and potential value as biomarkers of response to bariatric surgery.

## 1. Introduction

The concept of metabolic syndrome encompasses a wide range of metabolic abnormalities, including abdominal obesity, insulin resistance, hypertension and hyperlipidaemia, that result in a high risk of developing cardiovascular disease [1]. Although the number of different definitions of metabolic syndrome leads to discrepancies in its prevalence rates, the incidence and prevalence of metabolic syndrome go hand in hand with that of obesity and type 2 diabetes [2].

Lifestyle and environmental factors, in addition to genetic and epigenetic factors, are involved in the pathogenesis of metabolic syndrome [3]. Epigenetic mechanisms have been proposed to be a relevant factor involved in the development of metabolic diseases including metabolic syndrome, with epigenetics emerging as a promising tool for the management of metabolic diseases.

One of the most common epigenetic alterations associated with metabolic diseases is DNA methylation. Promoter methylation has usually been associated with a decrease in gene expression, whilst intragenic methylation has been related to increased gene expression [4].

Previous studies have investigated the relationship between epigenetic marks, DNA methylation-based, and metabolic syndrome as a whole or with separate components of the metabolic syndrome [5,6].

Furthermore, DNA methylation patterns respond to environmental factors, including weight loss intervention. Bariatric surgery is the most effective and durable treatment for weight loss and the amelioration of obesity-related comorbidities, including metabolic syndrome. Epigenetic factors have been suggested to be involved in the mechanisms related to metabolic improvement after bariatric surgery and the impact of bariatric surgery on DNA methylation levels has been shown in different tissues such as blood, adipose tissue, skeletal muscle and liver, among others [7]. Although DNA methylation changes have been associated with metabolic improvement observed after weight loss interventions [8,9], most of the studies have focused on evaluating the reversion of obesity-related epigenetic marks [10], and only a few studies have analyzed the potential role of epigenetic marks as predictors of the weight loss achievement and resolution of obesity-related comorbidities after bariatric surgery [11,12], although these studies have evaluated the methylation profiles of selected genes, warranting more studies to investigate the role of epigenetic marks in the prediction of bariatric surgery outcomes.

In a previous longitudinal study of the methylation status in a metabolically healthy obese population with a 11-year follow-up, we showed 26 differentially methylated CpG sites that could have a predictive role of the stability/worsening of metabolically healthy obese phenotype at long term [13]. The aim of the present study is to validate these epigenetics marks in a group of patients undergoing bariatric surgery and their association with the metabolic improvement observed after bariatric surgery, in order to identify those epigenetic marks that could predict the metabolic response to the bariatric surgery.

## 2. Results

The anthropometric and biochemical variables of the patients included in the study are shown in Table 1. Before surgery, triglyceride levels were significantly higher in the MetS non-responder group. There were no statistically significant differences between groups in the rest of variables studied at baseline. As was expected, patients improved their metabolic status 6 months after surgery; statistically significant differences between groups are shown in Table 1.

### 2.1. Validation of Differentially Methylated CpG Sites

**Figure 1:**
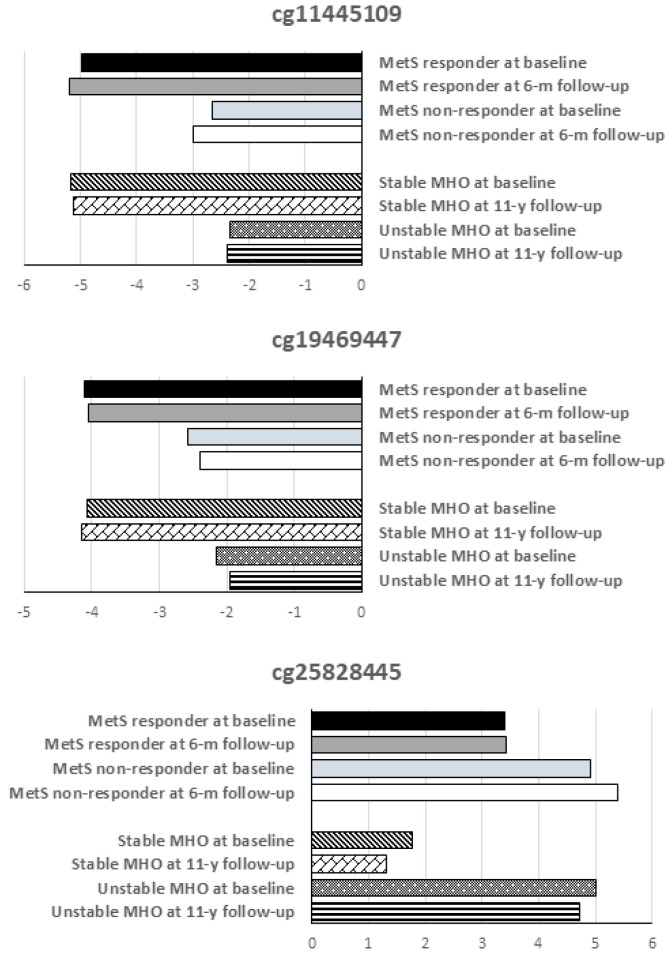
Methylation levels of the differentially methylated CpG sites. MetS responder: patients with metabolic syndrome who improved their metabolic status 6 months after bariatric surgery. MetS non-responder: patients with metabolic syndrome 6 months after surgery. Stable MHO: metabolically healthy obese patients during 11-year follow up. Unstable MHO: metabolically healthy obese patients who developed metabolic complications at 11-year follow-up.

Of the 26 significantly differentially methylated CpG sites previously described [13], three of them (cg11445109, cg19469447, cg25828445) were shown to be differentially methylated at baseline and 6 months after surgery. Both cg11445109 and cg19469447 (annotated to *CYP2E1* gene) were hypomethylated, with their methylation levels significantly lower in the MetS responder group compared to the MetS non-responder group both before surgery and six months after bariatric surgery, whilst cg25828445 (annotated to *NIFKP3* gene) was shown to be hypermethylated, with significantly higher methylation levels in the MeS non-responder group compared to the MetS responder group, both before surgery and 6 months after bariatric surgery (Figure 1). The methylation levels of the 26 CpG sites are shown in Supplementary Figure S1.

In a previous study based in a metabolically healthy obese population with a 11-year follow-up [13], both cg11445109 and cg19469447 were also hypomethylated, with their methylation levels being significantly lower in patients with metabolically healthy phenotype during 11-year follow-up compared to those who transitioned to unhealthy phenotype during the follow-up, whilst cg25828445 was significantly hypermethylated in those obese patients who transitioned to an unhealthy phenotype (Figure 1).

### 2.2. Differentially Methylated Genes

These 26 CpG sites were related to a total of 17 genes and two pseudogenes. A more in-depth analysis was performed on these genes. We collected all CpG sites in each of these genes from the UCSC genome browser and checked whether they were detected in the Methylation EPIC Bead. The detected CpG sites described in each gene were analyzed to investigate to what extent these genes presented multiple differentially methylated CpG sites. Fourteen of the nineteen genes identified showed multiple differentially methylated CpG sites. The gene with the highest percentage of methylated sites was *CYP2E1* (40%), followed by *HLA-DRB1* (33.33%) and *ZFPM2* (26.83%) (Table 2).

### 2.3. Associations between DNA Methylation and Other Anthropometric and Biochemical Variables

**Figure 2:**
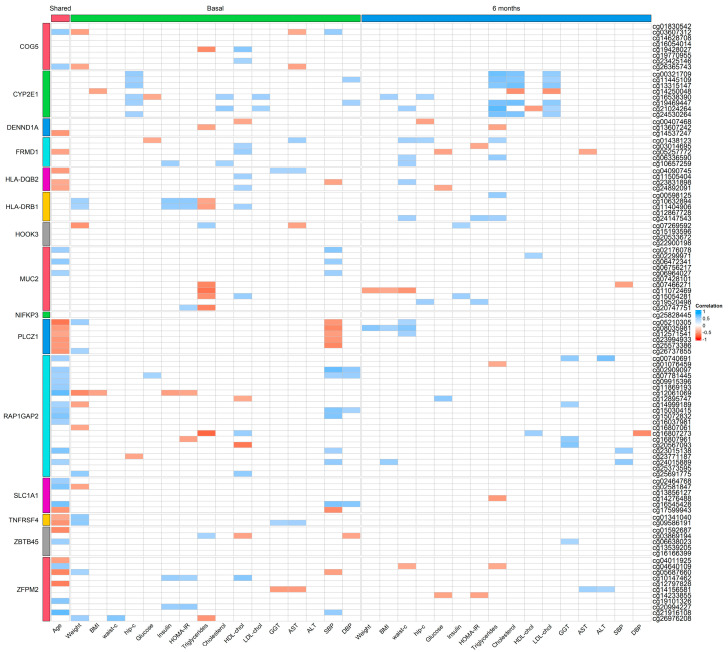
Heatmap correlation plot between methylation levels and anthropometric and biochemical variables at both study points.

We further analyzed the associations between anthropometric and biochemical variables and the methylation levels of differentially methylated CpG sites at both study points. Before surgery, cg11445109 and cg19469447 (annotated to *CYP2E1* gene) were correlated positively with hip circumference (r = 0.48; *p* = 0.04 and r = 0.48; *p* = 0.004, respectively) and diastolic blood pressure (r = 0.49; *p* = 0.003 and r = 0.47; *p* = 0.004, respectively). Six months after surgery, cg11445109 and cg19469447 (*CYP2E1* gene) were correlated positively with triglycerides (r = 0.61; *p* = 0.003 and r = 0.62; *p* = 0.003, respectively), cholesterol (r = 0.58; *p* = 0.007 and r = 0.65; *p* = 0.001, respectively) and LDL-cholesterol levels (r = 0.48; *p* = 0.03 and r = 0.54; *p* = 0.01, respectively). Cg25828445 (annotated to *NIFKP3* gene) showed no association with the anthropometric and biochemical variables included in the study (Figure 2).

The associations found with the rest of the differentially methylated CpG sites from each gene are shown in Figure 2. It should be highlighted that CpG sites annotated to *CYP2E1* (cg00321709, cg13315147, cg21024264 and cg24530264) correlated positively with triglycerides, cholesterol and LDL-cholesterol 6 months after bariatric surgery (Figure 2).

## 3. Discussion

In a previous study [13], we found 26 CpG sites associated with the stability/worsening of the metabolically healthy phenotype in a group of patients with obesity followed up for 11 years. In the present study, we studied these CpG sites in a group of patients with metabolic syndrome who underwent bariatric surgery and analyzed their association with metabolic outcomes observed after surgery. In this study, we confirm the potential role of cg11445109, cg19469447 and cg25828445 in the metabolic homeostasis and their association with metabolic health.

Our results showed that cg11445109 and cg19469447 (annotated to *CYP2E1* gene) were significantly more hypomethylated in patients who improved their metabolic status compared to those who remained with metabolic syndrome after bariatric surgery. Indeed, *CYP2E1* showed multiple differentially methylated sites in patients who improved their metabolic status after bariatric surgery compared to those who did not. In a previous study [13], we showed that the hypomethylation of both sites was associated with the stability of metabolically healthy phenotype in a group of patients followed up for 11 years.

*CYP2E1* is a member of the CYP superfamily, mainly expressed in hepatocytes, which is involved in the metabolism of several low-molecular weight xenobiotics such as ethanol or benzene, and endogenous compounds such as fatty acids and bile acids [14]. *CYP2E1* has been shown to be a relevant contributor to ROS production. In animal models, its expression has been associated with an increase in oxidative stress and apoptosis [15].

*CYP2E1* has been related to metabolic diseases; its activity has been shown to be increased in patients with T2DM and obesity [16,17] and several polymorphisms have been significantly associated with the risk of T2DM [18].

In animal models, *CYP2E1* deletion has been shown to induce adipose tissue browning, to increase whole-body energy expenditure and to improve glucose homeostasis [19]. *CYP2E1* deficiency has been suggested to activate PPARα, which is involved in lipid homeostasis and energy regulation [20]. Moreover, our results show that several CpG sites annotated to *CYP2E1* were correlated with markers of lipid levels at both study points. Previous studies have shown that overexpression of *CYP2E1* is associated with plasma lipid levels and liver steatosis [21]. These findings point out *CYP2E1* as a promising target to develop new strategies to fight against obesity and related diseases.

Our results show that cg25828445, annotated to *NIFKP3* gene, was significantly hypermethylated in patients who remained with metabolic syndrome after bariatric surgery. In a previous study [13], we showed that cg25828445 was significantly hypermethylated in those patients who transitioned to metabolically unhealthy phenotype in a group of patients followed up for 11 years.

To the best of our knowledge, there is little information about *NIFKP3*. *NIFKP3* is a processed pseudogene that is not encoded into a functional protein. Processed pseudogenes are a result of the reintegration of a gene due to retrotransposition. Pseudogenes have been suggested to have a regulatory role in the cells, and RNA transcripts of pseudogenes have been suggested to play a role as siRNA, miRNA-like and antisense RNA, which can regulate target gene expression [22].

The search for non-invasive biomarkers based on blood DNA methylation has increased in recent decades, becoming a huge challenge in metabolic diseases such as metabolic syndrome, a complex condition multifactorial in origin that involves multiple tissues and organs that are inaccessible without surgery. The search of biomarkers based on DNA methylation is an intricate task. DNA methylation is widely known to be influenced by genetic, physiological, and environmental factors. The gut microbiota, which has been shown to play an important role in host metabolism, and the use of chronic medication (i.e., metformin) are some of the factors whose actions have been suggested to be mediated by epigenetic modifications, which deserves further investigation.

This study highlights the potential role of DNA methylation as a predictive tool for metabolic outcomes after bariatric surgery. Further research is warranted to clarify the potential use of DNA methylation patterns as non-invasive biomarkers to predict metabolic improvement associated with bariatric surgery [11,23]. The identification of epigenetic biomarkers that might be useful in the prediction of metabolic improvement observed after bariatric surgery could help obesity management and development of appropriate treatment strategies.

Our study also presents some limitations. We could not perform RNA analysis to associate DNA methylation status to gene expression. Another limitation of the study is the small simple size. The use of peripherical blood could be considered a limitation of this study, although the main aim of this study is the identification of potential biomarkers. Whole blood is characterized by a high cellular heterogeneity, and methylation is a tissue-specific molecular mechanism. However, when it comes to the prediction role of a biomarker, this does not have to be involved in the causation of a disease, and may not have a mechanistic role in the disease.

However, as a strength of the study, we have confirmed the association between three epigenetic marks (cg11445109, cg19469447, cg25828445) and the metabolic status in a different population. Previously, we have shown the potential use of these CpG sites as markers of stability of metabolically healthy phenotype sites in patients with obesity followed up for 11 years with no weight loss intervention. On the other hand, in the present study, we have validated these CpG sites as potential markers of bariatric surgery response in patients with morbid obesity. 

## 4. Material and Methods

### 4.1. Subjects

This study was carried out at the Virgen de la Victoria University Hospital (Málaga, Spain). The inclusion criteria were patients with morbid obesity and metabolic syndrome who underwent laparoscopic sleeve gastrectomy procedure between January 2020 and December 2020 and attended both visits (before surgery and 6 months after surgery). Metabolic syndrome was established using the National Cholesterol Education Program (NCEP) Adult Treatment Panel III (NCEP ATPIII criteria) [24]. Patients were excluded if they had cardiovascular disease, acute inflammatory disease, infectious disease, or had complications after the surgery. From the patients who met the criteria, we selected the first 10 patients who were no longer patients with metabolic syndrome 6 months after the surgery (MetS responder group) and the first 10 patients who continued with metabolic syndrome 6 months after the surgery (MetS non-responder group).

Weight and height measurements were made before surgery and 6 months after surgery. Body mass index (BMI) was calculated as weight (kg)/height^2^ (m^2^). Blood pressure was measured twice using a sphygmomanometer with an interval of 5 min between measurements, and the average of the two measurements was calculated.

Blood samples were collected after a 10–12 h fast before surgery and 6 months after surgery. Serum was separated and immediately frozen at −80 °C. Blood glucose was measured using the glucose oxidase method (Bayer, Leverkusen, Germany). Total cholesterol, triglycerides and high-density lipoprotein cholesterol levels were measured using enzymatic methods. Insulin was determined using radioimmunoassay (DIASource ImmunoAssays SA, Nivelles, Belgium). The homeostasis model assessment of insulin resistance (HOMA-IR) was calculated as follows: fasting insulin (µIU/mL) × fasting glucose (mmol/L)/22.5.

### 4.2. DNA Methylation Assay

DNA was extracted from peripheral blood using the QIAmp DNA Blood Mini Kit (Qiagen, Hilden, Germany) following the manufacturer’s instructions. DNA concentration was quantified with a Qubit 3.0 Fluorometer (Thermo Fisher Scientific, Waltham, MA, USA) using Qubit dsDNA HS Assay Kit Fluorometer (Thermo Fisher Scientific, Waltham, MA, USA) After quantification, a total of 500 ng of genomic DNA was bisulfite-treated using a Zymo EZ-96 DNA Methylation™ Kit (Zymo Research Corp, Irvine, CA, USA) and was purified using a DNA-Clean-Up Kit (Zymo Research Corp, Irvine, CA, USA).

Over 850,000 methylation sites were interrogated with the Infinium Methylation EPIC Bead Chip Kit (Illumina, San Diego, CA, USA) following the Infinium HD Assay Methylation protocol, and raw data were obtained from iS (Illumina) software (v2.0).

### 4.3. Methylation Data Analysis

We used statistical programming language R 3.5.1 accessed on 1 March 2021 (https://www.r-project.org) to perform the methylation data analysis. Raw data files (idat files) were read with the minfi package [25] to calculate raw β-values. Normal-exponential out-of-band (NOOB) normalization [26] was used to correct the background. Probes located at sexual chromosomes or near SNPs were removed from the analysis. Low quality probes (those with a detection *p*-value > 0.01 in at least 10% of samples) were also removed.

Finally, beta-mixture quantile (BMIQ) normalization [27] was applied to correct for the two different bead designs in the microarrays. For the differential methylation analysis, we transformed β-values to M-values.

In the current study, we evaluate the methylation levels of those previously identified CpG sites and neighboring CpG sites related to the same genes (Supplementary Table S1) [13].

### 4.4. Statistical Analysis

Differences in anthropometric and biochemical variables were analyzed with the Mann–Whitney U test for continuous data and the chi-square test for categorial data using R software (3.5.1). Data were expressed as the mean ± standard deviation. Values were statistically significant when *p* < 0.05.

Differences between groups for each CpG site were analyzed using the Mann–Whitney U test. Correlations between DNA methylation levels, and anthropometric and biochemicals variables were analyzed using Spearman’s rank correlation coefficients.

## 5. Conclusions

In conclusion, we confirmed that cg11445109, cg19469447 and cg25828445 sites could have a potential role in the prediction of metabolic status. Identifying non-invasive blood-based biomarkers for metabolic syndrome may aid in diagnosing and screening those patients at risk of developing metabolic diseases. Importantly, these biomarkers may help in the management and treatment of patients with metabolic disease.

## Figures and Tables

**Table 1:** Anthropometric and biochemical variables of the patients included in the study.

	MetS Non-Responder at Baseline	MetS Responder at Baseline	MetS Non-Responder 6 m after Surgery	MetS Responder 6 m after Surgery
Sex (M/F)	1/9	3/7		
Age (years)	49.7 ± 10.29	47.7 ± 5.49		
Weight (kg)	126.45 ± 22.91	125.32 ± 20.56	101.29 ± 15.91	94.59 ± 12.35
BMI (kg/m^2^)	51.54 ± 10.34	45.9 ± 5.03	41.29 ± 7.25	34.73 ± 2.82 ^#^
Glucose (mg/dL)	113.4 ± 18.77	117.1 ± 28.85	96.60 ± 14.85	89.1 ± 7.92
Insulin (µUI/mL)	19.84 ± 9.01	27.91 ± 18.63	10.80 ± 2.78	11.39 ± 11.18
HOMA-IR	5.6 ± 2.95	8.21 ± 5.55	2.53 ± 0.63	2.54 ± 2.61
C-peptide (ng/mL)	4.59 ± 1.47	4.31 ± 1.37	3.14 ± 0.59	2.52 ± 0.81 ^#^
Cholesterol (mg/dL)	188.3 ± 25.18	175.6 ± 37.76	207.50 ± 37.24	174.3 ± 29.77 ^#^
Triglycerides (mg/dL)	197.3 ± 45.59	137.4 ± 70.45 *	153.60 ± 21.06	81.30 ± 19.39 ^#^
HDL-cholesterol (mg/dL)	38.6 ± 4.19	45.8 ± 8.95	43.20 ± 3.73	58.10 ± 12.83 ^#^
LDL-cholesterol (mg/dL)	111.18 ± 25.37	104.92 ± 31.39	131.26 ± 35.26	99.94 ± 24.88
AST (U/L)	28.5 ± 19.69	25.4 ± 13.38	17.56 ± 7.12	15.70 ± 3.77
ALT (U/L)	44.7 ± 26.06	39.3 ± 22.81	27.70 ± 8.90	21.40 ± 6.62
GGT (U/L)	46.8 ± 31.83	45.1 ± 17.46	36.00 ± 27.58	26.00 ± 14.53
SBP (mm Hg)	145.11 ± 24.4	137.8 ± 21.17	141.0 ± 17.45	124.4 ± 14.51 ^#^
DBP (mm Hg)	87.11 ± 13.14	83.4 ± 12.63	87.78 ± 11.55	78.6 ± 11.85
Diabetes treatment (y/n)	7/3	7/3	4/6	1/9
HTA treatment (y/n)	7/3	8/2	7/3	4/6

GGT: Gamma-glutamyl transferase. AST: aspartate aminotransferase. ALT: alanine aminotransferase. SBP: systolic blood pressure. DBP: diastolic blood pressure. * *p* < 0.05 between MetS non-responder and MetS responder group at baseline. ^#^ *p* < 0.05 between MetS non-responder and MetS responder group 6 months after surgery.

**Table 2:** CpG sites differentially methylated in the identified genes.

Gene	KEGG Orthology	Differentially Methylated CpG Sites	Total Number of CpG Sites	%
*RAP1GAP2*	Signaling and cellular processes	21	88	23.86
*MUC2*	Membrane trafficking	11	60	18.33
*ZFPM2*	Transcription factor	11	41	26.83
*COG5*	Membrane trafficking	8	63	12.70
*CYP2E1*	Metabolism	8	20	40.00
*PLCZ1*	Membrane trafficking	6	29	20.69
*SLC1A1*	Signaling and cellular processes	6	25	24.00
*FRMD1*	Signaling and cellular processes	5	42	11.90
*HLA-DRB1*	Signaling and cellular processes	5	15	33.33
*ZBTB45*	Transcription factor	5	27	18.52
*HLA-DQ2*	Signaling and cellular processes	4	49	8.16
*HOOK3*	Signaling and cellular processes. Membrane trafficking	4	35	11.43
*DENND1A*	Membrane trafficking	3	18	16.67
*TNFRSF4*	Signaling and cellular processes	2	13	15.38

## Data Availability

The data sets used and analyzed in this study are available from the corresponding authors on request.

## Supplementary Materials

The following supporting information can be downloaded at: https://www.mdpi.com/article/10.3390/ijms241914778/s1.
