# Supplementary material for: Epigenetic Marks as Predictors of Metabolic Response to Bariatric Surgery: Validation from an Epigenome Wide Association Study
Source: Int J Mol Sci. 2023 Sep 30;24(19):14778. doi: 10.3390/ijms241914778 (PMC10572880; doi:10.3390/ijms241914778)
Supplement: Supplementary file 1 [file ijms-24-14778-s001.zip › Suplementary Table S1.pdf]

Table S1: Significantly differentially methylated CpG sites selected accordingto their contribution to the PCA.

Cpg sites	Position	Relation	Gene	Island position	Location
**cg20707527**	8 q23.1	Genetic	ZFPM2	S_Shore	
**cg20381372**	19 q13.12	Intergenic	LOC100134317	S_Shelf	TSS1500
**cg20022036**	6 p21.32	Genetic	HLA-DRB1	N_shelf	Body
**cg15084585**	8 q23.1	Genetic	ZFPM2	S_Shore	
**cg23024343**	7 q22.3	Genetic	COG5	N_Shelf	Body
**cg20485733**	17 p13.3	Genetic	RAP1GAP2	Open Sea	Body
**cg20239921**	7 q 11.23	Genetic (Pseudogene)	DTX2P1-UPK3BP1-PMS2P11	Open Sea	Body
**cg07040661**	11 p15.5	Genetic	MUC2	N_Shelf	Body
**cg25458175**	19 q13.43	Genetic	ZBTB45	Island	Body
**cg07180897**	6 p21.32	Genetic	HLA-DQB2	N_Shore	Body
**cg08720517**	5 q 31.2	Genetic	PROB1	Island	1st Exon
**cg14073541**	9 q 33.3	Genetic	DENND1A	Open Sea	Body
**cg07458466**	12 p 12.2	Genetic	PLCZ1	Open sea	Body
**cg09642739**	8 p 11.21	Genetic	HOOK3	Open Sea	Body
**cg22949274**	11 q 23.1	Genetic	SDHD	S_Shelf	Body
**cg01437515**	1 p 36.33	Genetic	TNFRSF4		
**cg24840300**	19 p13.3	Genetic	PLPR3	Island	3'UTR
**cg25828445**	12 p13.31	Intergenic (Pseudogene)	NIFKP3	S_Shore	
**cg11445109**	10 q26.3	Genetic	CYP2E1	S_Shore	Body
**cg19469447**	10 q26.3	Genetic	CYP2E1	Island	Body
**cg17172308**	6 q27	Intergenic	FRMD1	S_Shelf	
**cg05473257**	10 q26.3	Genetic	CYP2E1	Island	Body
**cg14906510**	12 p 13.31	Genetic	NIFKP3	Island	
**cg05194426**	10 q26.3	Genetic	CYP2E1	S_Shore	Body
**cg23400446**	10 q26.3	Genetic	CYP2E1	Island	Body
**cg26805839**	9p24.2	Genetic	SLC1A1	Open Sea	Body
