# Supplementary figures and images for: Epigenetic Marks as Predictors of Metabolic Response to Bariatric Surgery: Validation from an Epigenome Wide Association Study
Source: Int J Mol Sci. 2023 Sep 30;24(19):14778. doi: 10.3390/ijms241914778 (PMC10572880; doi:10.3390/ijms241914778)

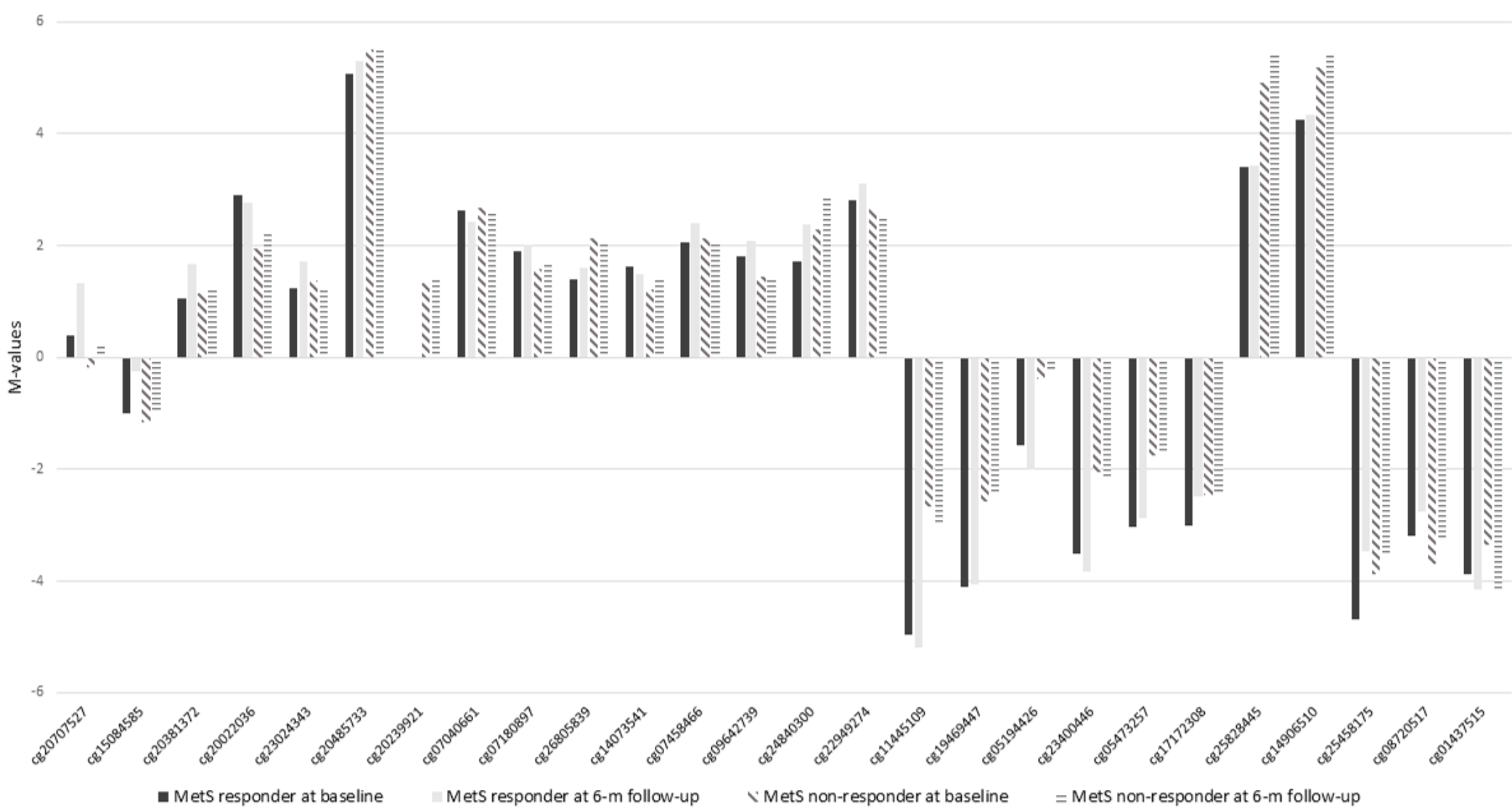

Supplement: Supplementary file 1 [file ijms-24-14778-s001.zip › Supplementary Figure S1.pdf]
